# DArT markers for the rye genome - genetic diversity and mapping

**DOI:** 10.1186/1471-2164-10-578

**Published:** 2009-12-03

**Authors:** Hanna Bolibok-Brągoszewska, Katarzyna Heller-Uszyńska, Peter Wenzl, Grzegorz Uszyński, Andrzej Kilian, Monika Rakoczy-Trojanowska

**Affiliations:** 1 Department of Plant Genetics, Breeding and Biotechnology, Warsaw University of Life Sciences (SGGW), 159 Nowoursynowska Str, 02-776 Warsaw, Poland; 2 Diversity Arrays Technology P/L, 1 Wilf Crane Crescent, Yarralumla, Canberra, ACT, 2600, Australia

## Abstract

**Background:**

Implementation of molecular breeding in rye (*Secale cereale *L.) improvement programs depends on the availability of high-density molecular linkage maps. However, the number of sequence-specific PCR-based markers available for the species is limited. Diversity Arrays Technology (DArT) is a microarray-based method allowing for detection of DNA polymorphism at several thousand loci in a single assay without relying on DNA sequence information. The objective of this study was the development and application of Diversity Arrays technology for rye.

**Results:**

Using the *Pst*I/*Taq*I method of complexity reduction we created a rye diversity panel from DNA of 16 rye varieties and 15 rye inbred lines, including parents of a mapping population consisting of 82 recombinant inbred lines. The usefulness of a wheat diversity panel for identification of DArT markers for rye was also demonstrated. We identified 1022 clones that were polymorphic in the genotyped ILs and varieties and 1965 clones that differentiated the parental lines L318 and L9 and segregated in the mapping population. Hierarchical clustering and ordination analysis were performed based on the 1022 DArT markers to reveal genetic relationships between the rye varieties and inbred lines included in the study. Chromosomal location of 1872 DArT markers was determined using wheat-rye addition lines and 1818 DArT markers (among them 1181 unique, non-cosegregating) were placed on a genetic linkage map of the cross L318 × L9, providing an average density of one unique marker every 2.68 cM. This is the most saturated rye linkage map based solely on transferable markers available at the moment, providing rye breeders and researches with a better choice of markers and a higher probability of finding polymorphic markers in the region of interest.

**Conclusion:**

The Diversity Arrays Technology can be efficiently and effectively used for rye genome analyses - assessment of genetic similarity and linkage mapping. The 11520-clone rye genotyping panel with several thousand markers with determined chromosomal location and accessible through an inexpensive genotyping service is a valuable resource for studies on rye genome organization and in molecular breeding of the species.

## Background

Winter rye (*Secale cereale *L.) is an important cereal crop in Central, Eastern and Northern Europe, mainly due to its exceptional ability to thrive and to produce high yields in adverse environmental conditions. Nutrient efficiency and tolerance of diseases exhibited by rye, allowing for a reduced usage of pesticides and fertilizers during production, increase its attractiveness for farmers and consumers. The recently better recognized dietary value of rye contributed to the noticeable growth of consumers' interest in rye products. Breeding progress in rye, however, is rather slow, as the traditional breeding is hampered by the outcrossing nature of this crop, self-incompatibility and occurrence of inbreeding depression.

Implementation of molecular breeding in rye improvement programs depends on the availability of high-density molecular linkage maps. Several genetic maps of rye have been published so far [1-4], but the possibilities of their practical application are rather limited, mainly due to an insufficient saturation. The average density of the most saturated rye linkage map published to date is 2.9 cM. However, there are seven gaps greater than 20 cM on the map [5]. The average map interval length on remaining rye maps exceeds 4.0 cM [2], in the majority of maps it is greater than 7 cM, i.e. 7.4 [6], 7.9 [4], 9.9 [7], 12.2 [8]. On the other hand, as the number of sequence-specific, PCR-based markers (SSRs and SCARs) available for the species was until recently below 300 [4,6,9-11], rye genetic maps were constructed predominantly with the use of usually poorly-transferable AFLP and RAPD markers, or labor intensive, time consuming and low-throughput RFLP markers. The highest number of sequence-specific, PCR-based markers on a rye linkage map equals 58 [4]. Lately, with the creation of BAC library specific for 1RS [12] and the development of 74 SSR markers for 1RS [13], a significant advance took place in rye genomics. However, the knowledge about the remaining part of the rye genome remains very limited.

The Diversity Arrays Technology (DArT) - a microarray, hybridization-based platform - has a capacity to deliver several thousand of sequence-specific markers without relying on sequence information [14]. To date, the performance of the method was validated in several species including cereals such as barley (*Hordeum vulgare *L.) [15], wheat (*Triticum aestivum *L.) [16] and sorghum (*Sorghum bicolor *(L.) Moench) [17]. The current list of species for which DArT arrays are available commercially as service is at http://www.diversityarrays.com and the most comprehensive review of the technology was presented by Kilian et al [18]. The technology finds increasing use in creating high density genetic maps [19] and association studies [20], but it is also expanding into the area of plant and animal biodiversity and population genetics [21,22]. The objective of this study was (i) to develop DArT markers for rye, (ii) to test their usefulness for assessing genetic similarity in rye inbred lines and varieties and (iii) to use DArT markers for constructing a high-density linkage map of the cross L318 × L9 [7].

## Results and Discussion

### Applicability of the PstI/TaqI complexity reduction method for the rye genome

Complexity reduction is a process, which reproducibly selects a defined fraction of genomic fragments and is a critical step in DArT. The *Pst*I/*Taq*I method is one of routinely used methods of genome complexity reduction in DArT assays and was shown to be superior to other method tested in wheat [16] and barley [15]. Consequently, this method was chosen for preparing targets form rye DNA in the preliminary experiments with a wheat diversity panel. The performance of *Pst*I/*Taq*I generated rye targets during hybridizations was satisfactory, as demonstrated by good values of quality parameters, and for this reason the *Pst*I/*Taq*I method of genome complexity reduction was used in all subsequent DArT analyses performed. Additionally, the preliminary hybridizations revealed wheat DArT markers that differentiated rye ILs. In total 768 candidate markers were identified on the wheat DArT array as polymorphic among the rye accessions tested, when the quality criteria of Q >77%, call rate > 80% and discordance <0.01 were applied. These clones ('wheat rearrayed for rye array' - two 384-well plates) were included in microarrays used for genotyping in genetic diversity and mapping analyses along with the clones from rye libraries. The utility of wheat DArT markers for rye genotyping is consistent with the previous data on cross transferability of molecular markers between wheat and rye - it was proven that wheat RFLPs [3] and SSRs [4] can be successfully used for rye genome analyses.

The experiments conducted with the rye genotyping array confirmed the usefulness of the *Pst*I/*Taq*I method for the analysis of the very large (ca. 8 Gbp), highly methylated and composed predominantly of repetitive sequences rye genome. It is clearly demonstrated by the proportion of clones displaying polymorphism in the materials genotyped. From the total of 1022 clones that were polymorphic in the genotyped ILs and varieties, 747 originated from the rye libraries, which corresponds to 12.2 polymorphism rate. The proportion of polymorphic clones in mapping experiments was slightly higher - 16.2 (from the total of 1965 clones that differentiated the parental lines L318 and L9 and segregated in the mapping population, 1742 originated from the 10752-clone rye library). However, those two numbers are not directly comparable as different marker selection criteria were used in diversity and mapping experiments. Moreover, the array extended with 4608 DNA clones from genetically diverse parental components of rye mapping populations was used for mapping. Those clones (mapping array 2.0) constituted 40% of the rye genotyping array 2.0 and 14.9% of them (686 markers) were polymorphic. Another 40% of the rye genotyping array 2.0 originated form DNA of parental lines of the population used in this study (mapping array 1.0) and 18.5% of them (851 markers) segregated in the RILs. The percentage of polymorphic markers observed for the diversity array during mapping experiments was 13.3 (205 markers). In the case of genetic diversity experiments the percentages of polymorphic markers were 13.1 (605 markers) and 9.2 (142 markers) for the arrays mapping 1.0 and diversity, respectively. The low number of polymorphic clones in the diversity array can be attributed to the low genetic diversity represented by the rye varieties used for library creation revealed in subsequent analyses. In general, the proportion of polymorphic markers in rye is higher than those reported for other cereals analyzed using DArT - wheat [16] and barley [15] - 9.4 and 10.4 respectively.

From 768 wheat DArT markers identified in the preliminary experiments, 275 differentiated ILs and varieties (35.8%), 223 were useful for mapping (29.0%) at P > 80%, Q>80% and a call rate of at least 90%.

### Genetic Diversity Analysis

**Figure 1:**
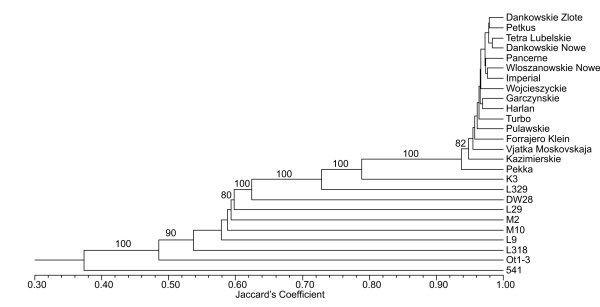
**UPGMA dendrogram based on the Jaccard's similarity matrix data obtained with 1022 DArT markers**. Bootstrap support values are shown if greater than 50%.

An UPGMA dendrogram based on the Jaccard's similarity matrix data obtained with 1022 DArT markers is shown in Figure 1. IL 541 separated from the remaining lines and varieties at about 38% similarity level. IL 541 is very sensitive to preharvest sprouting and originated from the cross involving the rye variety Smolickie and lines selected from the variety Kazimierskie [23]. While Kazimierskie was included in the study, to our knowledge no other IL analysed is related to Smolickie. In our opinion those are the two main factors that contributed to the observed outcome of the cluster analysis.

All the varieties analyzed were clustered together at ca. 94% similarity level. Very similar results were obtained by Ma et al [24], who analyzed spring and winter rye varieties using RAPD markers and reported clustering of winter varieties at similarity level slightly lower than 90%. Rye varieties are highly heterozygous [25]. Since DArT markers were scored in the described experiments as dominant markers (presence - score '1' - vs. absence - score '0' - of a marker in genomic representation hybridized to an array), it was not possible to distinguish between markers contained in heterozygous state (one copy of a target sequence per genomic representation) and homozygous markers (two copies per genomic representation). In addition, the genotypic and allelic frequencies are likely to vary among the varieties for a number of markers, and because DArT assays measure the differences in allelic frequencies well [26], the binarisation of signal intensity data by DArTsoft was likely seriously compromised, resulting in reduced frequency of markers discriminating varieties.

Genetic similarity (GS) coefficients for all 600 possible pairs of genotypes calculated on the basis of the Jaccard's coefficient ranged from 0.26 in the pair of ILs Ot 1-3 and 541 to 0.98 in each pair of varieties Dańkowskie Nowe and Dańkowskie Złote, Dańkowskie Nowe and Tetra, Dańkowskie Nowe and Włoszanowskie, Dańkowskie Nowe and Petkus. The ILs included in the experiment were analyzed previously with respect to genetic similarity based on data from 486 PCR-based markers [27]. The result of the 2-way Mantel test used for the comparison of similarity matrices from both studies was not statistically significant. This result is not surprising since DArT markers exploit a different source of polymorphism then the PCR- based markers used earlier. A poor correlation between estimates of similarity based on data derived using different markers systems was observed also in other studies [28]. At this point, it is not possible to judge, which markers - DArT or PCR-based - reflect the genetic relationships between rye genotypes more accurately, since only a small number of genotypes was common for both studies and for some of them a detailed prodigy information is not available. Moreover, pedigree information can be an insufficient criterion to conclude about genetic relationships between rye lines obtained using selfing. Since rye varieties are highly heterozygous, it can be expected that inbred lines selected from the same variety or cross will differ in a number of loci [29]. To resolve this issue an additional study of a larger set of rye genotypes involving an analysis of phenotypic features is needed.

The average GS coefficient values were 0.47 and 0.49 for DArT and PCR-based markers, respectively. Hence, in case of this study, the discriminating power of DArT markers didn't differ significantly from that of PCR-based markers. DArT markers, however, offer substantial advantages over PCR-based markers such as independence form gel electrophoresis and sequence information, transferability, reproducibility, automated scoring and high throughput, which allow obtaining more reliable results in a more cost effective way. These advantages make DArT a method of choice over serially produced low-plex markers for diversity studies [21].

**Figure 2:**
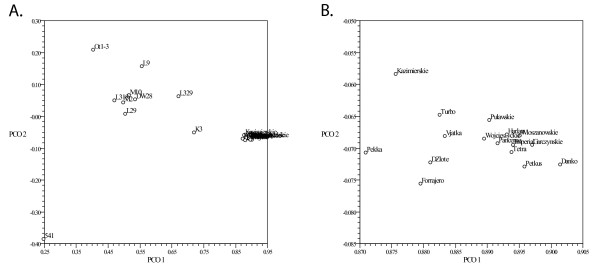
**Principal correspondence plot based on DArT data**. a. Patterns of relationships among 26 winter rye genotypes. b. Close up of the PCO plot section containing rye varieties. The first and the second principal axis explain 25.9% and 11.2% of the variation, respectively.

The principal correspondence plot generated from DArT data is shown in Figure 2. The first principal axis, explaining 25. 9% of the variation, differentiates rye varieties from ILs and resolves the differences within ILs, the second principal axis, explaining 11.2% of the variation, differentiates IL 541 from the remaining ILs and varieties.

### Chromosomal location of DArT markers

**Figure 3:**
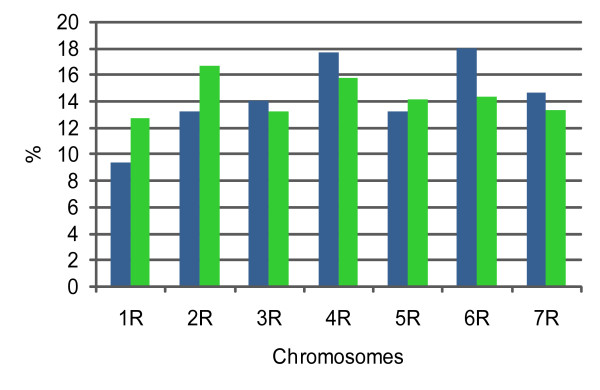
**Relation between DArT markers with determined chromosomal location and physical length of rye chromosomes**. Relation between the proportion of DArT markers localized on individual chromosomes using wheat-rye addition lines (blue bars) and the proportion of the physical length of the rye genome represented by individual chromosomes [30] (green bars).

Genotyping of wheat-rye addition lines on the rye genotyping array 2.0 revealed 1872 (16.3%) DArT markers that were present in genomic representation of only one of the addition lines (while marker quality criteria Q>80% and a call rate of at least 90% were applied), and therefore their chromosomal location could be determined. Proportions of DArT markers localized on individual chromosomes were not uniform and varied from 9.4% for the chromosome 1R (175 DArT markers) to 18.0% for the chromosome 6R (337 DArT markers). The result of the Spearman rank correlation analysis of these proportions with physical lengths of rye chromosomes [30] (Figure 3) was not statistically significant. From the DArT marker with determined chromosomal location, 367 (19.6%) displayed polymorphism in parental lines of mapping population and could be used as anchor loci during map construction.

### Genetic mapping of DArT markers

**Figure 4:**
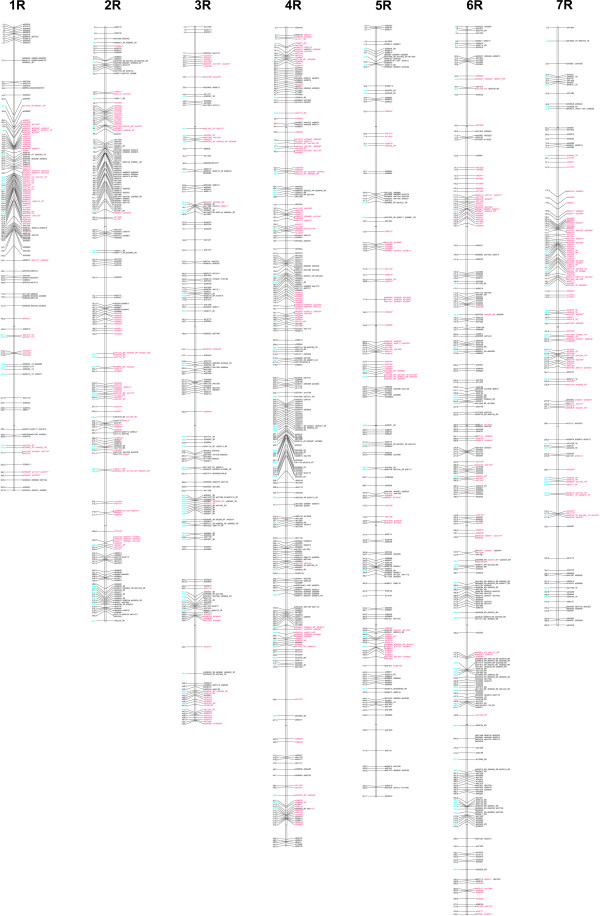
**Genetic linkage map of the cross L318xL9 containing 1818 loci**. Loci with distorted segregations at p < 0.01 are indicated by asterisks and shown in red. DArT markers with chromosomal location determined using addition lines are indicated by the symbol of the respective chromosome added after underscore, their map locations are shown in blue.

The Mendelian nature of DArT markers was demonstrated previously by Jacccoud at al. [14], Wenzl et al. [15] and Akbari et al. [16]. DArTs turned out to be highly efficient also in genetic mapping of rye. In total 1965 (17.1%) DArT markers differentiated ILs L9 and L318 and segregated in the RILs (under marker selection criteria described earlier). RECORD separated them into 43 linkage groups with marker number ranging from 2 (5 groups) to 182 (2 groups). Based on the presence of anchor loci (367 DArT and 20 SSR) the linkage groups were assembled using JoinMap 4 into 7 larger linkage groups representing rye chromosomes (Figure 4).

The resulting map contains 1818 markers. Among them 1181 loci (65%) were unique, non co-segregating with other markers. Number of markers per chromosome ranged from 178 (115 unique) for 3R to 380 (248 unique) for 4R with an average of 260 markers (169 unique) per chromosome. The map spans 3144.6 cM, providing an average density of one unique marker every 2.7 cM (Table 1). Markers were not evenly distributed along the chromosomes - clustering of markers was apparent in certain chromosome regions, on the other hand, 4 intervals that are longer than 20 cM long can be found on the map on the chromosomes 2R (20.5 cM), 4R (21.5 cM), 6R (20.1 cM) and 7R (20.7 cM). A consensus map of rye based on data from five mapping populations was published recently by Gustafson et al. [31]. The existence of five gaps of 10 cM or longer in the map was reported: four at the telomeric ends of the chromosomes - 1R (two gaps), 4R and 5R - and one in the middle of the short arm of the chromosome 6R. The complete lack of common markers between the L318 × L9 and the consensus map makes the comparison of gap locations impossible. We expect, however, that the ongoing creation of a DArT-based integrated map of several rye mapping population, including the DS2 × RXL10 cross (used also by Gustafson et al. [31]), will deliver the information necessary to answer, whether the particular gaps are population specific, result from limitation of the marker system used, or are conserved in rye.

**Table 1:** Characteristics of the L318 × L9 genetic map

	Chromosomes							Average	Total
	1R	2R	3R	4R	5R	6R	7R		
Number of loci	178	254	234	380	239	348	185	259.71	1818
Number of unique loci	115	167	153	248	145	224	129	168.71	1181
Number of loci with distorted segregation	83	88	52	120	88	81	121	90.43	663
Number of unique loci with distorted segregation	55	60	37	73	49	54	81	58.43	409
Percentage of loci with distorted segregation	46.63	34.65	22.22	31.58	36.82	23.28	65.41	37.23	34.82
Percentage of unique loci with distorted segregation	47.83	35.93	24.18	29.44	33.79	24.11	62.79	36.87	34.63
Number of DArT markers with chromosomal location determined using wheat-rye addition lines	25	44	59	53	31	89	36	48.14	337
Map length [cM]	301.9	387.1	452.4	533.3	501.7	578.7	389.5	449.2	3144.6
Map density based on unique loci	2.65	2.33	2.98	2.16	3.48	2.60	3.04	2.75	2.68

The length of the newly constructed L318 × L9 linkage map exceeded more than twice the length of the most saturated rye linkage map published to date, created by Bednarek et al. [5], based on analysis of a F_2 _population, which spanned 1386 cM and comprised 480 loci. This can be explained by map expansion due to the use of RILs, resulting from the multiple rounds of meiosis undergone [32,33]. A similar length relation between F_2 _and RIL-based genetic maps was observed earlier in maize [34]. As shown by Knox and Ellis [35], another factor that contributes to genetic map expansion is excess heterozygosity. An overrepresentation of heterozygots in L318 × L9 RILs was revealed in the earlier study of the population employing a larger number of SSR markers [7].

Of 1985 polymorphic markers, 729 (36.7%) deviated from the expected segregation ratio at the 1% level. Consistently with the results of previous mapping experiments conducted in rye on the same [7] and a different mapping population [8], the highest proportion of distorted unique markers was found on the chromosome 7R (62.8%). The percentage of distorted markers on remaining chromosomes varied from 24.1 (6R) to 47.8 (1R). Hackauf and Wehling [8] attributed the high percentage of distorted markers on the rye chromosome 7R maps to the existence of a locus governing zygotic selection on that chromosome. A high proportion of markers with segregation distortion observed on the chromosome 1R can be explained in a similar way as the self-incompability locus S is located there [36]. The segregation distortion is very likely to occur in a population of an out crossing crop suffering from inbreeding depression due to several cycles of selfing. Segregation distortion is a common phenomenon in rye [2,3,6,37] and in other plant species [34,38,39]. Importantly, it was shown not to influence the quality of mapping results both with simulated [40] and experimental data [34,41], even in the case of a RIL-based map with a higher proportion of distorted markers that reported in this study [34].

The DArT based L318 × L9 linkage map is the most saturated of the rye maps based solely on transferable markers available at the moment. In our opinion, the potential for its practical application is increased by the kind of the population used. RILs allow for repeated measurements and therefore for identifying loci influencing various traits of interest for breeders and researchers, but are relatively rarely used in rye genetics. The extent and uniformity of the genome coverage are still not sufficient for map based cloning and alignment of the (future) physical map. However, using the same array as used in this study for genotyping several other mapping populations, we identified over 5,000 markers segregating in at least one of these populations (data not reported). Integrating the information from several populations typed on the commercially available DArT array resulted in the construction of a high density consensus map of barley [19] and a similar strategy will be deployed to construct a 5,000+ DArT marker map of the rye genome. Having such a resource combined with a BAC anchoring strategy using DArT arrays (which was reported recently for wheat by Paux et al [42]), there is an opportunity to rapidly integrate genetic and physical mapping information for rye. Importantly, the relatively low level of marker redundancy observed in the mapping data points clearly to a potential for further array expansion using more candidate clones from additional varieties representing different germplasm pools. As some of the apparent marker redundancy in mapping data is likely to be due to genetic linkage (especially with relatively small RIL population size and non-random distribution along the chromosomes) rather than sequence redundancy, we can anticipate doubling the number of markers on the next generation array for the *Pst*I/*Taq*I representation. An even larger increase in DArT marker density can be achieved by deploying other methods of complexity reduction opening opportunities for rapid identification of tightly linked markers and providing a platform for positional cloning of rye genes.

## Conclusion

The presented study has demonstrated that the Diversity Arrays Technology can be efficiently and effectively used for the rye genome analyses - assessment of genetic similarity and linkage mapping. The 11520-clone rye genotyping panel, including 1872 DArT markers with chromosomal location determined using wheat-rye addition lines, and the DArT based genetic linkage map of the population L318 × L9 can be a valuable resource for studies on rye genome organization and in molecular breeding of the species. The array developed in the course of this study has been already applied with success to type a number of rye mapping populations.

## Methods

### Plant material and DNA isolation

DNA used in the experiments originated from 15 rye inbred lines (ILs): L9, L318, Ot 1-3, 541, L29, DW28, K3, L329, M2, M10, LM2020, RXL10, DS2, S76, S120, 16 rye varieties: Dańkowskie Złote, Kazimierskie, Turbo, Pancerne, Tetra Lubelskie, Dańkowskie Nowe, Włoszanowskie Nowe, Wojcieszyckie, Puławskie, Garczyńskie, Vjatka Moskovskaja, Imperial, Forrajero Klein, Harlan, Petkus, Pekka, 82 recombinant inbred lines (RILs) from the cross L318 × L9 and Chinese Spring-Imperial wheat - rye addition lines. DNA was isolated according to Murray and Thompson [43] from ca. 1 g of bulked leaf tissue of 4 weeks old greenhouse grown plants. Each IL and RIL was represented by 6 plants, each variety - by 16.

### DArT Analyses

#### Complexity reduction

Genomic representations for microarray preparation and for genotyping were prepared using the same complexity reduction method according to the protocol described by Yang et al. [44]. Briefly, ca. 100 ng of DNA were digested with restriction enzymes *Pst*I and *Taq*I (NEB). Simultaneously *Pst*I adapter was ligated. One μl of restriction/ligation reaction was used as a template in 50 μl amplification reaction using *Pst*I+0 primer. Sequences of adaptor and primer and cycling conditions are given in Yang et al. [44].

#### Microarray preparation

Preparation of Diversity Arrays Technology genomic libraries (diversity panels) and amplification of inserts from bacterial clones were carried out as described in Xia et al. [45].

A 1536-clone DArT genomic library ('diversity array'- four 384-well plates) was created from a mixture of DNA samples of 10 rye IL (without LM2020, RXL10, DS2, S76, S120) and all rye varieties included in the study. A 4608-clone library ('mapping array 1.0' - twelve 384-well plates) was prepared from a mixture of DNA of ILs L9 and L318 to increase the number of DArTs displaying polymorphism in RILs. Another 4608-clone library ('mapping array 2.0') was prepared from mixture of DNA samples of rye ILs 541, Ot1-3, LM2020, RXL10 and DS2.

Amplified inserts were precipitated, resuspended in spotting buffer DArT Spotter 2 (Wenzl et al., in preparation) and then printed on polylysine-coated slides using a MicroGrid II arrayer (Genomic Solutions, Lincoln, Neb.). For the first experimental trial, comprising genotyping of 10 rye ILs, rye varieties, and RILs from the cross L318 × L9, clones from arrays: diversity, wheat rearrayed for rye and mapping 1.0 (constituting together rye genotyping array 1.0) were printed in duplicate. For the second experimental trial, comprising genotyping of wheat-rye addition lines and RILs from the cross L318 × L9, clones from arrays: diversity, wheat rearrayed for rye, mapping 1.0 and mapping 2.0 (constituting together rye genotyping array 2.0) were printed without replication. After printing slides were put in water bath for 2 min at 95°C for DNA denaturation, then briefly immersed in 0.1 mM DTT, 0.1 mM EDTA solution and dried by centrifugation at 500 × g for 7 min.

#### Genotyping of DNA samples

Genomic representations of individual rye ILs, varieties, RILs and addition lines, prepared as described above were concentrated tenfold by precipitation with one volume of isopropanol, denatured for 3 min at 95°C and labeled with 1 μl 500 μM Cy3-labeled random decamers using the exo- Klenow fragment of *E. coli *DNA polymerase I (NEB).

Each labeled representation (target) was added to 50 μl of hybridization buffer (2 mM EDTA pH 8.0 solution containing 50 parts of ExpressHyb buffer (Clontech), 5 parts of 10 g × l-1 herring sperm DNA (Promega) and one part of the FAM-labeled polylinker fragment of the plasmid used for library preparation).

After denaturing targets were hybridized to microarrays overnight at 65°C. Subsequently slides were washed in 0.1 mM DTT, 1 × SSC, 0.1%SDS for 5 min, in 0.1 mM DTT, 1 × SSC for 5 min, in 0.1 mM DTT, 0.2 × SSC for 2 min and in 0.1 mM DTT, 0.02 × SSC for 30s. Immediately after washing slides were dried by centrifugation at 500 × g for 7 min at 30°C and then scanned with a fluorescent microarray scanner (Tecan LS300 scanner).

Batches of TIFF images pairs of individual slides were automatically analyzed by DArTsoft, a purpose-built software package developed at DArT P/L, to identify and score polymorphic markers as '1' - being present or '0' - being absent in the representation hybridized to a slide. Additionally, several parameters were computed by DArTsoft for evaluating the quality of markers, i.e. parameter Q (which measures the fraction of the of the relative target hybridization intensity as a percentage of the total variance), call rate (the percentage of DNA samples with defined '0' or '1' allele calls) and discordance (the fraction of concordant calls for replicate assays).

#### Testing the performance of the *Pst*I/*Taq*I method of genome complexity reduction

For testing the applicability of *Pst*I/*Taq*I method of complexity reduction for the DArT analysis of the rye genome, 12288 clones from a diversity panel constructed from wheat DNA [[16], http://www.diversityarrays.com] were printed onto microarrays. Targets prepared from DNA samples of 10 rye ILs and 16 varieties were hybridized to those microarrays in the manner described above.

### Genetic Diversity Analysis

The DArTsoft generated 0-1 scores were used to calculate a pair-wise genetic similarity matrix on the basis of the Jaccard's coefficient [46] using the NTSYS-pc, Version 2.1 [47] and then a cluster analysis was done to construct an UPGMA dendrogram. Cophenetic values were computed for the phenogram resulting in construction of cophenetic matrix. The goodness of fit of the phenogram was assessed by comparing the cophenetic matrix with the similarity matrix using the Mantel's statistic Z [48]. Bootstraping and calculation of the Jaccard's coefficient matrices from the resulting multiple datasets was performed with the PhylTools software [49]. A consensus tree was constructed with the Consense module of the PHYLIP V3.6 software package [50]. The Jaccard's similarity matrix generated from marker scores was also used for Principal Coordinate Analysis (PCO) to visualize the genetic relationships between the rye genotypes. Only clones with Q > 80% and a call rate of at least 90% were included in the analyses.

### Map Construction

Marker scores obtained after hybridizations of RILs and the parental lines L9 and L318 to the rye genotyping array 1.0 and the rye genotyping array 2.0 were combined and DArT markers with P values from both experiments greater than 80% and additionally markers with P value from one experiment above 85% were chosen for map construction. The scores of polymorphic DArT markers were converted into genotype codes according to the scores of the parents. RECORD [51] was used to determine linkage groups and the order of markers within linkage groups. Linkage groups were assembled into chromosomes based on the presence of anchor loci - SSR (Table 2) and DArT. The segregations of SSR anchor loci were determined in an earlier study of the same mapping population [7]. Order of markers within chromosomes was established applying the maximum likelihood algorithm of JoinMap 4.0 [52] with the use of marker orders determined by RECORD. Graphical genotypes were inspected visually to verify the ordering of the markers. Genetic maps of the chromosomes were calculated for the established marker orders with the help of MAPMAKER/EXP 3.0 [53]. Recombination fractions were converted to cM with the Kosambi mapping function. The graphical representation of the map was drawn using MapChart software [54].

**Table 2:** SSR anchor loci used during map construction

Marker	Chromosome	Reference
SCM9	1R	[3]
SCM0171	1R	[7]
SCM0107	1R	[8]
SCM0127	1R	[8]
SCM43	2R	[6]
SCM0023	2R	[7]
SCM0041	2R	[7]
SCM0095	3R	[7]
SCM0162	3R	[8]
SCM0047	4R	[8]
SCM0139	4R	[7]
SCM0029	5R	[7]
SCM0098	5R	[7]
SCM0172	5R	[7]
SCM0168	6R	[7]
SCM180	6R	[6]
SCM86	7R	[6]
SCM0063	7R	[7]

## Data deposition

All DArT marker scores reported in this paper were deposited in the GrainGenes database http://wheat.pw.usda.gov/GG2/index.shtml and they can be retrieved using the link: http://wheat.pw.usda.gov/cgi-bin/graingenes/browse.cgi?class=allele;query=DArT*

## Competing interests

Employees of DArT P/L co-authoring this paper (Andrzej Kilian, Katarzyna Heller-Uszyńska, Peter Wenzl and Grzegorz Uszyński) may benefit financially from this work.

## Authors' contributions

HBB participated in molecular analyses, genetic mapping and the design of the study, performed hierarchical clustering and ordination analysis and drafted the manuscript, KHU participated in molecular and DArTsoft analyses, PW participated in genetic mapping, GU participated in DArTsoft data analyses and downstream processing, AK participated in the design and coordination of the study, guided array development and data production and helped to draft the manuscript, MRT conceived of the study, participated in its design and coordination and helped to draft the manuscript. All authors read and approved the final manuscript.
